# The Athlete-IQ: Evaluating iron knowledge of female athletes and staff; with insights into practices among dietitians and iron experts

**DOI:** 10.1016/j.jsampl.2025.100118

**Published:** 2025-10-22

**Authors:** Michael Pengelly, Kate Pumpa, David B. Pyne, Naroa Etxebarria

**Affiliations:** a University of Canberra, Research Institute for Sport and Exercise (UCRISE), Canberra, ACT, 2617, Australia; b University College Dublin, Health Sciences Centre, 4 Stillorgan Rd, Belfield, Dublin 4, Ireland

**Keywords:** Survey, Performance, Health, Mineral, Deficiency, Nutritional practices

## Abstract

**Background:**

Iron deficiency (ID) is common in female athletes, with variable diagnostic criteria and supplementation protocols used to determine deficient athletes. Understanding iron rich food sources is fundamental to optimal nutrition intake. Purpose: Our aim was to characterise the iron knowledge of female athletes and affiliated staff and explore factors influencing iron deficiency practices among dietitians and iron experts (sports physiologists and iron researchers).

**Methods:**

A cross-sectional design was employed using a web-based iron questionnaire (adapted from previously validated questionnaires). Part One consisted of 16 items (11 iron questions) and was completed by female athletes and staff. Participants achieving a score >75 ​% (percentage of correct answers) were deemed to have adequate iron knowledge. Part Two consisted of 22 items (14 on iron deficiency practice) and was completed by dietitians and iron experts. Descriptive statistics were used for multiple choice questions and open responses were coded thematically.

**Results:**

Sixty-two participants (athletes n ​= ​42; staff n ​= ​20) across 10 sports completed Part One. Iron knowledge scores ranged from 21 ​% to 88 ​% among athletes (46 ​% ​± ​13 ​%) and staff (60 ​% ​± ​16 ​%). Twenty-eight practitioners (sports dietitians: n ​= ​14; other professions: n ​= ​14) across 28 sports completed Part Two. Between 10 ​% and 56 ​% of practitioners applied established criteria for diagnosing iron deficiency. Supplementation protocols varied between 20 and 100 ​mg/day or bi-daily of elemental iron for earlier stages of iron deficiency increasing to 60–200 ​mg/day or bi-daily for anaemic athletes.

**Conclusion:**

Iron knowledge is highly variable in female athletes and club staff. Teams and organisations should promote valid nutrition resources when access to a sports dietitian is not feasible. Practitioners may adopt heightened criteria to categorise iron deficiency but should adjust individual thresholds based on factors underpinning fluctuations in iron status.

## Graphical abstract

**Figure:**
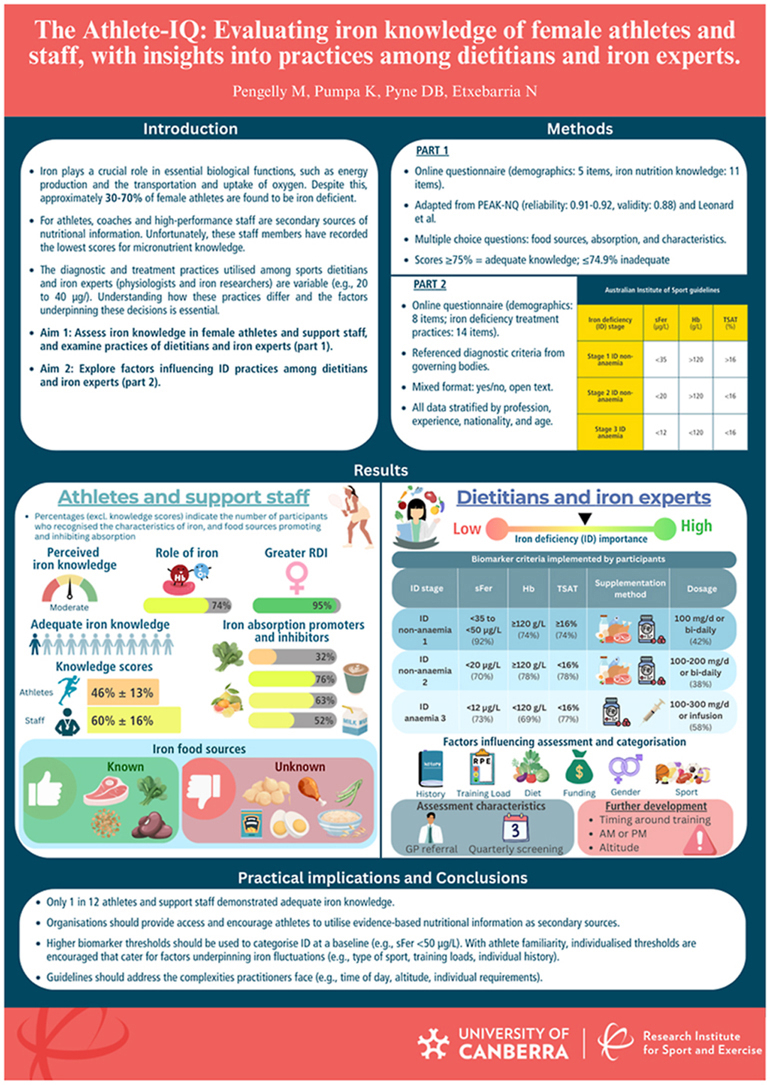

**Key points**

• Teams and organisations should provide access to valid and quality nutrition sources of information to mitigate the influence of peripheral sources that may compromise an athlete's health.
• Heightened absolute criterion to categorise ID should be utilised as a baseline (especially for less experienced practitioners). However, with experience and familiarity, practitioners can adopt a dynamic individualised approach that caters for factors underpinning fluctuations in iron status including gender, sport, and training loads.
• Advanced guidelines addressing the categorisation and treatment of ID in athletes including the timing of supplementation relative to training sessions, time of day, and sporting requirements are necessary to aid practitioners.

## 1 Background

Sports performance is enhanced with adequate nutrition intake of both macro- and micronutrients that facilitate key physiological processes (e.g., energy production, transportation and uptake of oxygen). Iron is a micronutrient with key biological implications on successful sports performance and health including energy production, red blood cell production, and transportation and uptake of oxygen. However, iron deficiency remains one of the most prevalent micronutrient deficiencies globally, especially among female athletes [1,2]. Iron losses are exacerbated among eumenorrheic female athletes [1]. In addition, there are other factors contributing to iron losses in female athletes beyond menstruation including low dietary intake, disordered eating, and relative energy deficiency in sports [3]. For example, 60 ​% of female triathletes and long-distance runners presented as iron deficient (ID) compared to 40 ​% of male triathletes and 30 ​% of male long-distance runners [1]. Given the heightened prevalence, it is pertinent for female athletes to have adequate iron knowledge to satisfy the higher recommended dietary intake. Maximising iron consumption may offset losses experienced as a female athlete that could negatively affect their training and competition performance.

Many female athletes often compete in a part-time capacity with financial restrictions and therefore have limited access to sports dietitians [4]. Beyond sports dietitians, a primary source of nutrition information for athletes are the affiliated staff, including coaches and other performance staff (e.g., strength and conditioning coaches, physiotherapists, and performance analysts) [5,6]. These sources are in addition to peripheral influences including social media, fellow athletes, parents and social networks [5,6]. While sports physicians understand the categorisation and treatment of clinical iron deficiency, other staff may have varying knowledge about adequate iron sources and the mechanisms leading to iron loss or compromised metabolism. No research has explored in detail the iron knowledge among female athletes and a broad range of staff. Multiple studies have reported poor nutrition knowledge in female athletes with micronutrient knowledge scores among the lowest across several nutrition themes (∼40 ​%) [[7], [8], [9], [10]]. Similarly, macro- and micro-nutrient knowledge scores were lowest among assessed nutrition themes in key staff (coaches, athletic trainers) with scores ranging between 52 ​% and 71 ​%, but adequate (≥75 ​%) in strength and conditioning coaches (76 ​%) [5]. Characterising the range and variability of nutrition advice is important to evaluate professional practices and the development of educational materials and programs.

Treating iron deficiency among athletes is complicated given the varying protocols used by dietitians and iron experts (physiologists and researchers) to correct it. Despite the introduction of best-practice guidelines from governing bodies [11], it is unclear what criteria practitioners use for supplementation and how this differs by profession or location. For example, previous research studies have utilised serum ferritin (sFer) cut-off values of up to <40 ​μg/L [12] to categorise iron deficiency, with supplementation doses ranging from 16 to 100 ​mg/day of elemental iron [13,14]. Therefore, the aims of this study were two-fold. The first aim was to quantify the iron knowledge of female athletes, and affiliated staff (i.e., coaches, performance staff, physicians) coaches, sports physicians, and performance staff, and secondly, to explore the factors underpinning the categorisation and treatment of iron deficiency in athletes by dietitians and iron experts.

## 2 Methods

### 2.1 Experimental design and subjects

This study employed a cross-sectional design using a web-based questionnaire and was conducted in two parts on the Qualtrics platform (Qualtrics Ltd; access date: May 2025). Part One (Online supplementary file 1) consisted of an iron knowledge questionnaire completed by high-level (tiers 3 to 5, national to world class irrespective of age) [15] female athletes and affiliated staff (i.e., coaches, high-performance staff, and sports physicians). For this study, ‘staff’ will reference coaches, performance staff, and physicians collectively. Male athletes and dietitians were excluded from Part One. Participants were recruited through sporting organisations associated with the University of Canberra and the Female Performance and Health Initiative newsletter distributed by the Australian Institute of Sport. Part Two (Online supplementary file 2) of the questionnaire focused on the practices and treatment of iron deficiency and was completed by sports dietitians and iron experts (sports physiologists and iron researchers). Sports dietitians were invited to participate through Sports Dietitians Australia and professionals in nutrition for exercise and sport via advertisement of the project. sports physiologists and iron researchers who have published studies on iron deficiency and athletic performance were invited to participate in the study via email. These professions were included given their expertise in iron metabolism and athletic performance. Survey responses for both parts were collected between July 2023 and May 2024. This study was conducted according to the guidelines laid down in the Declaration of Helsinki and all procedures involving human subjects were approved by the University of Canberra Human Research Ethics Committee (Approval number 11908). All participants were provided with detailed information about the study design and potential benefits before providing their informed consent, which was a prerequisite for advancing to the questionnaire.

### 2.2 Instrument

Part One of the iron questionnaire included a demographics section with 5 items, followed by 11 items examining iron nutrition knowledge adapted from the PEAKS-NQ (reliability: 0.91–0.92, validity ​= ​0.88) [16] and Leonard, Chalmers, Collins, Patterson [17] studies. The demographics section collected information on gender, age, profession including ‘athlete’, and sport. The iron nutrition knowledge section comprised a mix of multiple-choice and true-false questions, covering topics such as food sources, absorption, and characteristics. Each correct response was allocated one point, with a maximum possible score of 24. Knowledge scores were calculated by dividing the number of correct answers by the total number of items, with a score of 75 ​% or above indicating adequate iron knowledge, and scores below 75 ​% indicating inadequate iron knowledge [5]. After each question, participants rated their confidence in their answers using a 4-point Likert scale (1 ​= ​not at all confident, 2 ​= ​not very confident, 3 ​= ​somewhat confident, 4 ​= ​very confident). Confidence scores were analysed separately from correct and incorrect answers.

Part Two of the iron questionnaire consisted of 22 multiple choice and short-answer questions, including a demographics section with 8 items, followed by 14 items exploring the identification, treatment, and management of ID in athletes among sports dietitians and iron experts. The demographics section gathered data on gender, country of residence, qualification, profession experience, and athlete demographics (tier, sex, sport) they primarily work with.

### 2.3 Statistical analysis

A sufficient sample size for each part of the questionnaire was determined to be 66 participants (based on an accessible population of ∼200). This estimate was based on a 95 ​% confidence level, a 10 ​% margin of error, and a conservative 33 ​% response rate using Raosoft [18] sample size calculator. For both parts of the questionnaire, demographic variables were grouped based on response distributions to create approximately even group sizes for comparison. Given that Part Two of the questionnaire only had 28 completed responses, and that sub-group analyses for both parts were smaller than the a priori target of 66 participants, these analyses were performed for exploratory purposes only.

Basic descriptive statistics were used to present demographic data, knowledge scores, and confidence scores across both parts of the study. Demographic data and knowledge scores were expressed as percentages, while confidence scores were reported as averages. The Mann–Whitney U test was employed to examine differences between demographic variables based on profession (athlete or staff) and experience (<5 years or ≥6 years) in Part One. Data analysis was conducted using SPSS (IBM SPSS Statistics, version 28, Armonk, NY, USA), with the alpha level set at 0.05 for all analyses. In Part Two, basic descriptive statistics were also used for multiple-choice questions (questions 10, 12, 13, 15, 16, 19, and 21) to identify trends between demographic variables based on country of residence (Australasia or international), experience (<10 years or ≥11 years), and profession (sports dietitian or other professionals such as academics, sports physiologists, and registered dietitians). For open-ended questions, responses were thematically coded using phenomenological analysis and presented as percentages to reflect the frequency of each theme across all responses.

## 3 Results

Sixty-one participants (athletes n ​= ​42; -staff n ​= ​19) across 10 sports (60 ​% from women's Australian Rules Football) completed Part One of the questionnaire. The percentage of correct answers for each question ranged between 11 ​% and 95 ​% (Table 1, Table 2). The average confidence score for all correct answers ranged between 1.8 and 3.5 (1 ​= ​not at all confident to 4 ​= ​very confident; Table 1, Table 2)**.** Knowledge scores for all participants ranged between 21 ​% and 88 ​%. Knowledge scores were higher (*p* ​= ​0.002) for staff (60 ​% ​± ​16 ​%; mean ​± ​SD) than athletes (46 ​% ​± ​13 ​%), but not based on experience (i.e.,≤5 or≥6 years; *p* ​= ​0.825). Knowledge scores for correct answers relating to iron characteristics per question in Part One, with the associated confidence score (mean and SD) are presented in Table 1, Table 2

**Table 1:** Knowledge scores of athletes and affiliated staff for correct answers relating to iron characteristics per question in Part One, with the associated confidence score (mean ​± ​SD).

	All		Profession					Experience				
			Athlete		Staff			<5 years		>6 years		
n ​=	62		42		20			27		35		
**Chose the most important role in the body for iron.**	**KS**	$\bar{x}$ ± **SD**	**KS**	$\bar{x}$ ± **SD**	**KS**	$\bar{x}$ ± **SD**	***P* value**	**KS**	$\bar{x}$ ± **SD**	**KS**	$\bar{x}$ ± **SD**	***P* value**
Delivery of oxygen to muscles	74 ​%	3.1 ​± ​0.6	67 ​%	2.9 ​± ​0.6	90 ​%	3.3 ​± ​0.6	0.110	74 ​%	3.0 ​± ​0.7	74 ​%	3.1 ​± ​0.6	0.869
**Which of the following are features of haem iron (as opposed to non-haem iron)?**												
Iron is attached to a protein	29 ​%	2.5 ​± ​0.9	14 ​%	2.0 ​± ​0.8	60 ​%	2.8 ​± ​0.8	<0.010	15 ​%	2.3 ​± ​0.4	40 ​%	2.6 ​± ​1.0	0.151
More easily absorbed by humans	35 ​%	2.5 ​± ​0.9	21 ​%	2.0 ​± ​0.7	65 ​%	2.9 ​± ​0.8		30 ​%	2.1 ​± ​0.8	40 ​%	3.1 ​± ​0.5	
Found in animal foods	35 ​%	2.8 ​± ​0.8	17 ​%	2.1 ​± ​0.8	75 ​%	3.1 ​± ​0.6		30 ​%	2.1 ​± ​0.8	40 ​%	3.1 ​± ​0.5	
**For adults aged 19**–**30 years, is the recommended daily intake of iron higher for biological men or women?**												
Women	95 ​%	3.0 ​± ​0.9	95 ​%	2.9 ​± ​0.9	95 ​%	3.3 ​± ​0.6	0.968	96 ​%	3.0 ​± ​0.9	94 ​%	3.1 ​± ​0.9	0.717
**Why do you believe this is the case? (re. previous question)**												
Menstrual blood loss	94 ​%	2.6 ​± ​0.7	95 ​%	2.5 ​± ​0.7	90 ​%	2.8 ​± ​0.6	0.932	100 ​%	2.5 ​± ​0.7	89 ​%	2.6 ​± ​0.7	0.218
To build iron stores during pregnancy	24 ​%	2.1 ​± ​0.6	21 ​%	2.0 ​± ​0.5	30 ​%	2.3 ​± ​0.7		33 ​%	2.2 ​± ​0.6	17 ​%	2.0 ​± ​0.6	
Losses during childbirth	29 ​%	2.6 ​± ​0.6	21 ​%	2.4 ​± ​0.5	45 ​%	2.7 ​± ​0.7		26 ​%	2.4 ​± ​0.5	31 ​%	2.6 ​± ​0.6	

KS, knowledge score; $\bar{x}$, mean; SD, standard deviation.

All p-values represent group differences.

**Table 2:** Knowledge scores of athletes and affiliated staff for correct answers relating to nutrition sources and dietary factors per question in Part One, with the associated confidence score (mean ​± ​SD).

	All		Profession					Experience				
			Athlete		Staff			<5 years		>6 years		
n ​=	62		42		20			27		35		
**Which of these foods are a good source of iron?**	**KS**	$\bar{x}$ ± **SD**	**KS**	$\bar{x}$ ± **SD**	**KS**	$\bar{x}$ ± **SD**	***P* value**	**KS**	$\bar{x}$ ± **SD**	**KS**	$\bar{x}$ ± **SD**	***P* value**
Lamb	82 ​%	2.7 ​± ​0.7	76 ​%	2.6 ​± ​0.7	95 ​%	3.0 ​± ​0.6	0.047	78 ​%	2.7 ​± ​0.6	86 ​%	2.8 ​± ​0.6	0.848
Chickpeas	52 ​%	2.6 ​± ​0.6	48 ​%	2.4 ​± ​0.6	60 ​%	2.9 ​± ​0.5		48 ​%	2.5 ​± ​0.6	54 ​%	2.7 ​± ​0.6	
Green beans	42 ​%	2.5 ​± ​0.6	38 ​%	2.4 ​± ​0.5	50 ​%	2.8 ​± ​0.6		52 ​%	2.6 ​± ​0.5	34 ​%	2.5 ​± ​0.5	
Chicken	23 ​%	2.9 ​± ​0.8	17 ​%	2.3 ​± ​0.7	35 ​%	3.4 ​± ​0.5		15 ​%	2.0 ​± ​0.7	29 ​%	3.2 ​± ​0.7	
**Which of these vegetarian foods are a good source of iron?**												
Spinach	79 ​%	2.7 ​± ​0.7	79 ​%	2.7 ​± ​0.7	80 ​%	2.8 ​± ​0.6	0.152	85 ​%	2.7 ​± ​0.7	74 ​%	2.7 ​± ​0.7	0.875
Lentils	73 ​%	2.6 ​± ​0.7	71 ​%	2.4 ​± ​0.6	75 ​%	2.8 ​± ​0.7		85 ​%	2.4 ​± ​0.6	63 ​%	2.7 ​± ​0.7	
Baked beans	42 ​%	2.8 ​± ​0.6	29 ​%	2.5 ​± ​0.5	70 ​%	3.0 ​± ​0.5		30 ​%	2.6 ​± ​0.5	51 ​%	2.8 ​± ​0.6	
Eggs	19 ​%	2.5 ​± ​0.8	24 ​%	2.3 ​± ​0.6	10 ​%	3.5 ​± ​0.5		11 ​%	2.3 ​± ​0.5	26 ​%	2.6 ​± ​0.8	
**Which of the following foods has the most iron?**												
100 ​g lean grilled steak (trimmed)	81 ​%	2.9 ​± ​0.7	83 ​%	2.8 ​± ​0.7	75 ​%	3.2 ​± ​0.7	0.441	74 ​%	2.8 ​± ​0.7	86 ​%	3.0 ​± ​0.7	0.254
**Which of the following foods has the most iron?**												
1 cup (260 ​g) cooked porridge (made with full fat milk)	11 ​%	2.1 ​± ​0.8	12 ​%	1.8 ​± ​0.7	10 ​%	3.0 ​± ​0.0	0.826	15 ​%	2.3 ​± ​0.8	9 ​%	2.0 ​± ​0.8	0.445
**You have eaten chickpeas at lunch. What foods would you add to improve iron absorption of your meal?**												
Citrus fruits (e.g., oranges, mandarins)	63 ​%	3.1 ​± ​0.9	57 ​%	3.0 ​± ​0.8	75 ​%	3.3 ​± ​0.9	0.227	56 ​%	2.9 ​± ​0.9	69 ​%	3.2 ​± ​0.8	0.352
Spinach	32 ​%	2.4 ​± ​0.8	33 ​%	2.2 ​± ​0.9	30 ​%	2.7 ​± ​0.5		37 ​%	2.0 ​± ​0.9	29 ​%	2.7 ​± ​0.5	
**Red meat has more than double the amount of iron of chicken or fish (gram for gram).**												
True	82 ​%	2.4 ​± ​0.7	86 ​%	2.3 ​± ​0.7	75 ​%	2.7 ​± ​0.7	0.306	85 ​%	2.3 ​± ​0.7	80 ​%	2.5 ​± ​0.8	0.599
**The absorption of iron from meat foods is increased by vitamin C consumed in the same meal.**												
False	16 ​%	2.3 ​± ​1.0	12 ​%	2.4 ​± ​1.2	25 ​%	2.2 ​± ​0.7	0.194	15 ​%	2.3 ​± ​0.4	17 ​%	2.3 ​± ​1.2	0.806
**Tea and coffee inhibit iron absorption.**												
True	76 ​%	2.6 ​± ​0.6	69 ​%	2.4 ​± ​0.6	90 ​%	2.7 ​± ​0.7	0.074	74 ​%	2.6 ​± ​0.5	77 ​%	2.5 ​± ​0.7	0.781
**Calcium enhances iron absorption.**												
False	48 ​%	2.8 ​± ​0.9	36 ​%	2.7 ​± ​1.0	75 ​%	2.9 ​± ​0.4	0.004	52 ​%	2.7 ​± ​0.5	45 ​%	2.9 ​± ​1.1	0.634

KS, knowledge score; $\bar{x}$, mean; SD, standard deviation.

All p-values represent group differences.

**Fig. 1:**
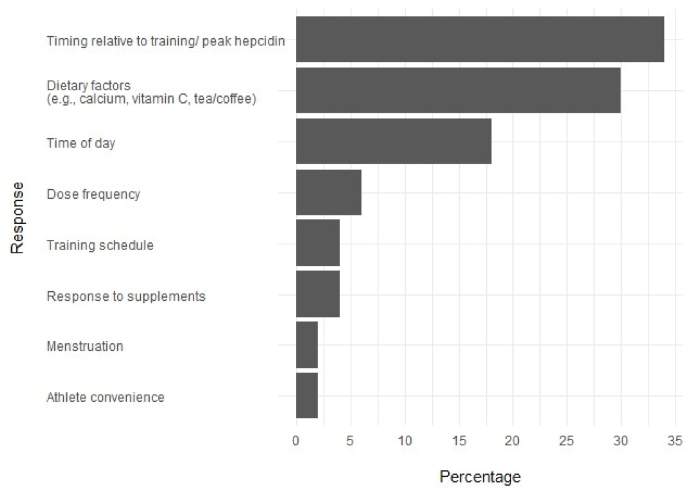
Responses to the question “Do you consider the athlete's training schedule and meal composition when recommending the timing of supplement ingestion? If yes, what recommendations to do you provide to athletes.” displayed as a frequency following thematic coding by employing phenomenology.

**Fig. 2:**
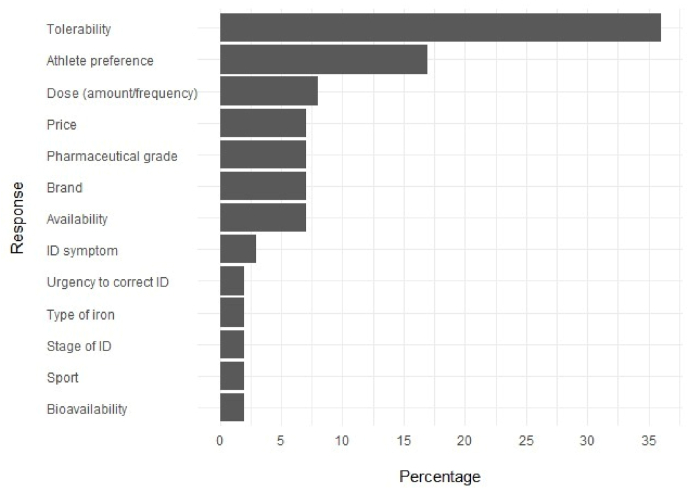
Responses to the question “What factors influence the type of supplement recommended” displayed as a frequency following thematic coding by employing phenomenology.

There was a 53 ​% completion rate (n ​= ​53) for Part Two of the questionnaire with 28 practitioners (sports dietitians n ​= ​14; other professions n ​= ​14) across 28 sports (40 ​% from aerobic-dominant sports) answering all questions. Iron assessment characteristics of these practitioners are detailed in Table III. Practitioners reported reassessing iron status in athletes approximately every 3 months. The minimum panel required to assess iron status, including sFer, haemoglobin concentration (Hb), and transferrin saturation (TSAT), was used consistently by most practitioners (64 ​%–100 ​%). Practitioners generally opposed the referenced criteria (Table IV) for determining stages of ID, with only 10 ​%–56 ​% agreeing with the prescribed thresholds (Table IV). Supplementation protocols varied between doses of 20–100 ​mg/d of elemental iron for earlier stages of ID, increasing the dose to 60–200 ​mg/d for anaemic athletes. Key considerations for advising iron supplementation included timing relative to training and dietary factors among others (Fig. 1). Factors influencing the recommended supplementation included tolerability and athlete preference among others (Fig. 2). Mann–Whitney U analysis revealed non-significant differences in the level of agreeance on assessment biomarkers and characteristics with consideration to location, experience, and profession.

**Table 3:** Iron status assessment characteristics of practitioners in part two.

	All	Country		*P* value	Experience		P value	Qualification		*P* value
		Australia/New Zealand	International		<10 years	>11 years		Diet.	Other	
n ​=	28	19	9		11	17		14	14	
**Iron status assessment frequency (all athletes)**										
Bi-annually	21 ​%	16 ​%	33 ​%	0.885	–	–	0.711	29 ​%	–	0.454
Only when suspected ID	21 ​%	21 ​%	22 ​%		36 ​%	24 ​%		21 ​%	21 ​%	
Other	36 ​%	42 ​%	22 ​%		27 ​%	41 ​%		36 ​%	36 ​%	
**Iron status reassessment frequency (iron deficient athletes)**										
Every 3 months	75 ​%	84 ​%	56 ​%	0.223	90 ​%	67 ​%	0.711	86 ​%	64 ​%	0.401
**Blood markers used to assess iron deficiency**										
sFer	100 ​%	100 ​%	100 ​%		100 ​%	100 ​%		100 ​%	100 ​%	
Hb	86 ​%	84 ​%	89 ​%		82 ​%	88 ​%		86 ​%	86 ​%	
TSAT	71 ​%	74 ​%	67 ​%		64 ​%	76 ​%		71 ​%	71 ​%	
sTfR	43 ​%	21 ​%	89 ​%		27 ​%	53 ​%		21 ​%	64 ​%	

Diet., sports dietitians; sFer, serum ferritin; Hb, haemoglobin concentration; TSAT*,* transferrin saturation; sTfR, soluble transferrin receptor.

Note: Only answers that received a response rate of >20 ​% are displayed.

All p-values represent group differences.

**Table 4:** Level of agreement among sports dietitians and iron experts on the referenced [11], to define each stage of iron deficiency in part two of the questionnaire.

		All	Country		*P* value	Experience		P value	Qualification		*P* value
			Australia/New Zealand	International		<10 years	>11 years		Diet.	Other	
**Referenced criteria**	n ​=	28	19	9		11	17		14	14	

**Stage 1**	**Stage 1 iron deficiency non-anemia**										

<35 ​μg/L	sFer	33 ​%	22 ​%	56 ​%	0.176	20 ​%	41 ​%	0.386	29 ​%	38 ​%	0.685
≥120 ​g/L	Hb	26 ​%	28 ​%	22 ​%	0.820	20 ​%	29 ​%	0.711	29 ​%	15 ​%	0.375
≥16 ​%	TSAT	26 ​%	22 ​%	33 ​%	0.668	20 ​%	29 ​%	0.711	29 ​%	23 ​%	0.830

**Stage 2**	**Stage 2 iron deficiency non-anemia**										

<20 ​μg/L	sFer	30 ​%	17 ​%	56 ​%	0.106	10 ​%	41 ​%	0.187	21 ​%	38 ​%	0.458
≥120 ​g/L	Hb	22 ​%	22 ​%	22 ​%	1.000	10 ​%	29 ​%	0.414	29 ​%	15 ​%	0.583
<16 ​%	TSAT	22 ​%	17 ​%	33 ​%	0.495	10 ​%	29 ​%	0.414	21 ​%	23 ​%	0.943

**Stage 3**	**Stage 3 iron deficiency anemia**										

<12 ​μg/L	sFer	27 ​%	18 ​%	44 ​%	0.253	20 ​%	31 ​%	0.711	23 ​%	31 ​%	0.685
<120 ​g/L	Hb	31 ​%	24 ​%	44 ​%	0.375	20 ​%	38 ​%	0.537	31 ​%	31 ​%	0.943
<16 ​%	TSAT	23 ​%	18 ​%	33 ​%	0.495	20 ​%	25 ​%	0.902	23 ​%	23 ​%	0.943

Diet., sports dietitians; sFer, serum ferritin; Hb, haemoglobin concentration; TSAT, transferrin saturation.

All p-values represent group differences.

## 4 Discussion

### 4.1 Iron knowledge

It is imperative for female athletes and support staff to understand the role and implications of iron deficiency among female athletes. Despite this, iron knowledge among female athlete and some support staff is limited. While it is well understood that iron requirements are higher in females (than males), athletes and staff (excluding sports dietitians) appear to have limited knowledge on iron-rich food sources. This knowledge gap likely hinders efforts to prevent declines in iron status female athletes may experience, which are exacerbated by training (e.g., heightened training loads) [19] and other personal factors (e.g., menstrual bleeding) [20].

Athletes often refer to coaches, performance staff, and teammates for nutrition information [5,6]. However, relying on non-dietitian sources increases the likelihood of nutrition misinformation. In this study, only five participants (athletes: n ​= ​1; staff: n ​= ​4) demonstrated adequate iron knowledge, despite two-thirds indicating a moderate understanding of iron— findings consistent with previous research indicating inadequate micro- and macronutrient knowledge by athletes and affiliated staff [5,[7], [8], [9], [10]]. The limited knowledge may reflect a lack of formal nutrition education among support staff, coupled with athletes' continued reliance on them as sources of nutrition information over sports dietitians, thereby amplifying the potential for misinformation that may negatively impact an athlete's health, wellbeing, and performance. For example, while most staff acknowledged calcium inhibits iron absorption, two-thirds of athletes incorrectly indicated calcium enhances iron absorption.

Limited knowledge of iron-rich foods among athletes and staff may hinder management of low or suboptimal iron levels. While most participants identified red meat, spinach, and legumes as iron rich sources, fewer (20 ​%–50 ​%) recognised alternatives including chickpeas, green beans, baked beans, and eggs. Without a broad understanding of iron-rich foods, female athletes may struggle to meet the recommended dietary intake of 18 ​mg/day; particularly vegetarian or vegan athletes given the lower bioavailability of non-haem iron [21]. The limited iron knowledge among athletes and staff suggests they do not fully understand the nutritional quality of their meals. For example, only 20 ​% recognised eggs as an iron-rich food despite eggs being more commonly consumed. Similarly, although 80 ​% identified spinach as iron-rich, 70 ​% were unaware of its role in enhancing iron absorption due to its high vitamin C content.

Improving iron knowledge could enable more well-informed food choices and help athletes meet daily requirements. Where access to sports dietitians is limited, organisations should provide access to credible resources (e.g., Australian Institute of Sport infographics) [22] to reduce reliance on peripheral sources and ensure athletes are equipped with the knowledge necessary to safeguard health, wellbeing, and performance.

### 4.2 Iron deficiency - treatment practice

ID is a diverse issue, exacerbated by the lack of consistent global guidelines, leading to substantial differences in treatment practices. Despite these variations, it is pertinent practitioners utilize evidence-based criteria as a guideline while adopting an individualized approach tailored to each athlete.

When assessing iron status, there appears to be a general global consensus that modified criteria beyond standard clinical thresholds are required for athletes (e.g., stage one ID non-anaemia (IDNA1): sFer <35 ​μg/L). Elevated biomarker thresholds account for exercise-related iron losses such as gastrointestinal bleeding and hepcidin regulation [23,24]. Elite female athletes can experience 25–40 ​% decrements in sFer during high training loads despite adequate dietary intake (i.e., 18 ​mg/d of elemental iron) [19], supporting the need for athlete-specific criteria. Indeed, 92 ​% of practitioners (n ​= ​24) use sFer <35 ​μg/L or higher (up to <50 ​μg/L) to categorize IDNA1, while Hb and TSAT generally align with clinical thresholds (i.e., Hb: >120 ​g/L; TSAT: >16 ​%). Despite ∼55 ​% of internationals initially indicating their criteria differed, only one reported using clinical thresholds (sFer <30 ​μg/L) for the categorization of IDNA1; the remainder applied equal or higher (sFer <45 ​μg/L) sFer criterion (n ​= ​6) or did not specify (n ​= ​2). More experienced practitioners (>11 years) were more likely to use heightened criteria (e.g., sFer <50 ​μg/L) or individualized thresholds that account for factors including the athlete's size, sport, lifestyle, and history. For example, ID prevalence varies markedly between female long-distance runners and triathletes (∼60 ​%) [1] compared to rugby sevens athletes (∼30 ​%)^2^**.** While referenced criteria are a useful guideline (particularly for less experienced practitioners), a dynamic approach tailored to the athlete may be necessary as practitioner familiarity increases.

Discrepancies were evident in iron supplementation protocols. For IDNA1, prescribed doses were up to five-fold higher with dosages ranging between 20 (n ​= ​1) to 100 (n ​= ​7) mg/d or bi-daily. Similar variability was observed for stage two ID non-anaemia (IDNA2; 25–100 ​mg/d or bi-daily) and stage three ID anaemia (IDA3; 60–300 ​mg/d or bi-daily). These differences did not favour a demographic (i.e., profession, experience, country of residence) but likely reflect the individualized considerations essential for tailoring supplementation to each athlete. When advising supplementation protocols, practitioners prioritised individual tolerability and preference. For example, one practitioner reduced the dose from 100 ​mg/d to 60 ​mg bi-daily in response to gastrointestinal distress. This conservative approach may explain the wide range of recommended dosages observed (i.e., 20–100 ​mg/d for IDNA1 and IDNA2; 60–300 ​mg/d for IDA3). Beyond tolerability and preference, factors such as sport and urgency to correct the ID emphasize the need for dynamic individualised protocols.

Best practice recommendations were more variable when considering training and altitude. Around 60 ​% of practitioners accounted for post-exercise hepcidin peaks and 35 ​% for time of day, with advice split between supplementing before (55 ​%) or after training (45 ​%). While the optimal absorption window is ∼30 ​min either side of training [25], post-training recommendations ranged widely from 30 ​min to 3 ​h. Hepcidin is an iron-regulatory hormone that down-regulates intestinal iron absorption by binding to and degrading the solitary cellular iron exporter, ferroportin [26]. Hepcidin expression is modulated by inflammation, with synthesis increasing following a rise in interleukin-6 (an inflammatory cytokine) and peaking between 3- and 6-h post-exercise [27,28]. Its diurnal variation, which peaks in the afternoon (∼16:00) [29], may explain why practitioners often recommend morning supplementation. Although previous studies indicated higher iron absorption in the morning [30], recent findings suggest little difference by time of day [31]. Currently, the most appropriate timing appears to be within the 30 ​min window before or after training [25]. However, this intricacy remains secondary to the to the overriding priority, athlete compliance.

Uncertainty around treating ID in sport is increased at altitude. Practitioners used a wide range of ferritin cut-offs (<35 ​μg/L to <100 ​μg/L), with some practitioners avoiding taking iron-deficient athlete altogether. This conservative approach reflects the increased erythropoietic demand at altitude thus potentially precluding iron-deficient athletes with low sFer levels (e.g., <30 ​μg/L). Indeed, red blood cell volume failed to increase in endurance athletes following 4 weeks at altitude when pre-altitude sFer levels were inadequate and subsequently could not tolerate the three to five-fold increase in erythropoiesis at altitude [32,33]. Consistent with this, the Australian Institute of Sport advises against altitude exposure for iron-deficient athletes and recommends 100 ​mg/day or bi-daily supplementation for those with an sFer <100 ​μg/L, starting 2 weeks before and continuing throughout exposure. Notably, the varying sFer criteria for altitude supplementation (i.e., <35 ​μg/L to <100 ​μg/L) are primarily reported by Australian practitioners.

Responses on altitude treatment were often superficial, reflecting the lack of a global framework to guide supplementation during different training phases. While protocols at sea level are well-documented, those for altitude remain in their infancy. Among documented practices, sFer and haemoglobin mass increase by 37 ​% and 4 ​%, respectively, in endurance athletes (sFer 25 ​± ​7 ​μg/L) following the ingestion of 205 ​mg/day of elemental iron combined with 2–4 weeks of moderate altitude exposure [34]. Greater haemoglobin mass improvements have been reported in athletes with an sFer <20 ​μg/L ingesting 210 ​mg/d of elemental iron, whereas no improvement occurs when pre-altitude sFer is ​< ​35 ​μg/L and supplementation is limited to 105 ​mg/d. It appears supplementation of ∼200 ​mg/d of elemental iron may be most effective for iron-deficient athletes (sFer <35 ​μg/L), while those with higher sFer levels (>35 ​μg/L) should consult a sports physician and sports dietitian for best practice advice. The uncertainty surrounding iron treatment protocols underscores the need for comprehensive guidelines. Such guidelines should address the complexities practitioners face including timing of supplementation (relative to training and time of day) and specific recommendations for altitude conditions. Therefore, future research should continue to explore differing iron supplementation protocols addressing timing and at altitude to establish best practice guidelines.

### 4.3 Limitations

This study has several limitations. The sample was disproportionately represented by Australian rules football athletes and Australasian practitioners, which may reduce external validity and generalisability beyond these cohorts. Additionally, although the questionnaire was adapted from the PEAKS-NQ [16] and Leonard, Chalmers, Collins, Patterson [17] study, reliability and construct validity was not assessed for this modified version. Finally, as with all declarative, self-reported data, participation may have been influenced by an individual's interest in iron and the web-based format imposed additional constraints (e.g., participants could not save and resume the questionnaire which may have contributed to the 53 ​% completion rate and limiting participation to those with internet access and English literacy).

## 5 Conclusion

Iron knowledge among female athletes and club staff—excluding dietitians—remains highly variable. To mitigate the influence of peripheral sources, teams and organisations should facilitate access to valid nutrition sources when accessing a sports dietitian is not feasible. For practice, applying heightened absolute criteria (e.g., sFer <35 ​μg/L) appears warranted initially, but thresholds should remain flexible to account for individual fluctuations in iron status. However, practitioners should remain flexible with individual thresholds based on factors underpinning fluctuations in iron status. Future guidelines should address supplementation timing and altitude exposure.

## Author’s contributions

MP, KP, DP and NE conceptualized the study and methodology; MP was responsible for the project administration; MP conducted the investigation. MP, KP, DP and NE contributed to the formal analysis; MP, KP, DP and NE wrote the draft manuscript, and MP, KP, DP and NE contributed to the final manuscript.

## Protocol

There is no pre-registered protocol relevant to this study to declare.

## Funding sources

This research did not receive any specific grant from funding agencies in the public, commercial, or not-for-profit sectors.

## Declaration of competing interest

The authors declare that they have no known competing financial interests or personal relationships that could have appeared to influence the work reported in this paper.
