# Supplementary material for: The Athlete-IQ: Evaluating iron knowledge of female athletes and staff; with insights into practices among dietitians and iron experts
Source: JSAMS Plus. 2025 Oct 22;6:100118. doi: 10.1016/j.jsampl.2025.100118 (PMC13008435; doi:10.1016/j.jsampl.2025.100118)
Supplement: Multimedia component 1 [file mmc1.pdf]

Tools Saved at 12:23 PM Draft Preview Publish

The Athlete-IQ: Section 1

ExpertReview score Fair

Introduction

Q0

The Athlete-IQ will ask for a small amount of personal information about your gender, age, sport, and number of years spent competing or practicing as either a high-level female athlete, coach, sports physician, or performance staff member (e.g., strength and conditioning coach). The Athlete-IQ will then ask you a series of questions about your knowledge of iron. There are five (5) questions relating to demographic information and eleven (11) questions relating to iron knowledge. You will be asked to indicate your level of confidence in each answer for questions 6 to 16 on a 4-point Likert scale (1= not at all confident; 2= not very confident; 3= somewhat confident; 4= very confident). This questionnaire will take approximately 10 minutes to complete.

This information will examine iron knowledge among high-level female athletes, coaching staff, sports physicians, and performance staff. A report will be provided to you concluding the study summarising the responses to each question.

Your survey data will be securely stored in the University of Canberra's Qualtrics server and treated with absolute confidentiality. In any manuscripts, reports, or other publications resulting from this study, you will not be able to be identified. In compliance with the university protocol, all data collected from the surveys will be stored at the University of Canberra for five years and then destroyed.

- ○ I agree that I am providing informed consent to participate in this project.
- ○ I do not agree that I am providing informed consent to participate in this project (please close the web page).

Import from library Add new question

Add Block

Demographics

Q1

1. Please indicate your gender.

- ○ Female
- ○ Male
- ○ Non-binary / third gender
- ○ Prefer not to say

Q2 *

2. Please indicate your age.

- ○ <15 years old
- ○ 16-20 years old
- ○ 21-25 years old
- ○ 26-30 years old
- ○ 31-35 years old
- ○ >36 years old

Q3 *

3. Please indicate your profession.

- ○ Athlete
- ○ Coach
- ○ Sports physician
- ○ Performance staff

Q4

4. Please indicate your sport (if you compete or work across multiple sports, please indicate your primary sport).

Q5 *

5. Please indicate the number of years you have competed or practiced as a high-level athlete, coach, physician, or performance staff member.

- ○ <1 year
- ○ 2-3 years
- ○ 4-5 years
- ○ 6-10 years
- ○ 11+ years

Page Break

Import from library Add new question

Add Block

Iron knowledge

Q6

6. Do you think your knowledge of dietary iron is? (Please tick one option only).

- ○ Low
- ○ Moderate
- ○ High

Q7a

7. Chose the most important role in the body for iron.

- ○ Muscle growth and repair
- ○ Strong bones and teeth
- ○ Delivery of oxygen to muscles
- ○ A healthy digestive system

Q7b

7b. Please indicate your level of confidence in the selected answer above.

- ○ 1= not at all confident
- ○ 2= not very confident
- ○ 3= somewhat confident
- ○ 4= very confident

Q8a

8a. Which of these foods are a good source of iron?

- ☐ Chickpeas
- ☐ Apples
- ☐ Lamb
- ☐ Milk
- ☐ Green beans
- ☐ Chicken
- ☐ Not sure

Q8b

8b. Please indicate your level of confidence in the selected answer above.

- ○ 1= not at all confident
- ○ 2= not very confident
- ○ 3= somewhat confident
- ○ 4= very confident

Q9a

9a. Which of these vegetarian foods are a good source of iron?

- ☐ Onions
- ☐ Baked beans
- ☐ Lentils
- ☐ Vegetable stock
- ☐ Spinach
- ☐ Eggs
- ☐ Cheese
- ☐ Not sure

Q9b

9b. Please indicate your level of confidence in the selected answer above.

- ○ 1= not at all confident
- ○ 2= not very confident
- ○ 3= somewhat confident
- ○ 4= very confident

Q10a

10a. Which of the following foods has the most iron?

- ○ ½ cup (60g) cooked spinach
- ○ 100g lean grilled steak (trimmed)
- ○ 1 cup (190g) cooked white rice
- ○ Not sure

Q10b

10b. Please indicate your level of confidence in the selected answer above.

- ○ 1= not at all confident
- ○ 2= not very confident
- ○ 3= somewhat confident
- ○ 4= very confident

Q11a

11a. Which of the following foods has the most iron?

- ○ ½ cup (50g) cooked kidney beans
- ○ 1 cup (260g) cooked porridge (made with full fat milk)
- ○ 1 boiled egg
- ○ Not sure

Q11b

11b. Please indicate your level of confidence in the selected answer above.

- ○ 1= not at all confident
- ○ 2= not very confident
- ○ 3= somewhat confident
- ○ 4= very confident

Q12a *

12a. You have eaten chickpeas at lunch. What foods would you add to improve iron absorption of your meal? (Select all answers below that apply).

- ☐ Citrus fruits (e.g., oranges, mandarins)
- ☐ Tea and coffee
- ☐ Spinach
- ☐ Dairy products
- ☐ Not sure

Q12b

Please indicate your level of confidence in the selected answer above.

- ○ 1= not at all confident
- ○ 2= not very confident
- ○ 3= somewhat confident
- ○ 4= very confident

Q13a *

13a. Which of the following statements are true regarding iron in the diet?

	True	False
a) Red meat has more than double the amount of iron of chicken or fish (gram for gram)	○	○
b) The absorption of iron from meat foods is increased by Vitamin C consumed in the same meal	○	○
c) Tea and coffee inhibit iron absorption	○	○
d) Calcium enhances iron absorption	○	○

Q13b

13b. Please indicate your level of confidence in the selected answer above.

- ○ 1= not at all confident
- ○ 2= not very confident
- ○ 3= somewhat confident
- ○ 4= very confident

Q14a *

14a. Which of the following are features of haem iron (as opposed to non-haem iron)? (Select all answers below that apply).

- ☐ Iron is attached to a protein
- ☐ Found in plant foods
- ☐ Found in animal foods
- ☐ More easily absorbed by humans
- ☐ Less easily absorbed by humans
- ☐ Don't know

Q14b

14b. Please indicate your level of confidence in the selected answer above.

- ○ 1= not at all confident
- ○ 2= not very confident
- ○ 3= somewhat confident
- ○ 4= very confident

Q15a *

15a. For adults aged 19–30 years, is the recommended daily intake of iron higher for biological men or women?

- ○ Men
- ○ Women
- ○ Not sure

Q15b

15b. Please indicate your level of confidence in the selected answer above.

- ○ 1= not at all confident
- ○ 2= not very confident
- ○ 3= somewhat confident
- ○ 4= very confident

Q16a

16a. Why do you believe this is the case? (Select all answers below that apply).

- ☐ Menstrual blood loss
- ☐ Losses during childbirth
- ☐ Greater concentration of testosterone enhancing iron metabolism
- ☐ Improve sperm motility
- ☐ Lower concentration of growth hormone
- ☐ To build iron stores during pregnancy
- ☐ Not sure

Q16b

16b. Please indicate your level of confidence in the selected answer above.

- ○ 1= not at all confident
- ○ 2= not very confident
- ○ 3= somewhat confident
- ○ 4= very confident

Import from library Add new question

Add Block

End of Survey

We thank you for your time spent taking this survey.

Your response has been recorded.
