# Supplementary material for: The Athlete-IQ: Evaluating iron knowledge of female athletes and staff; with insights into practices among dietitians and iron experts
Source: JSAMS Plus. 2025 Oct 22;6:100118. doi: 10.1016/j.jsampl.2025.100118 (PMC13008435; doi:10.1016/j.jsampl.2025.100118)
Supplement: Multimedia component 2 [file mmc2.pdf]

Tools Saved at 12:22 PM Published

Preview Publish

The Athlete-IQ: Section 2

Expert

Introduction

Q0

The Athlete-IQ will ask for a small amount of personal information about your gender, qualification, number of years spent practicing in your profession, primary sports you work with, and basic demographic information relating to each sport. The Athlete-IQ will then ask you a series of questions about your knowledge and expertise relating to the assessment of iron ar treatment. There are eight (8) questions relating to demographic information, nine (9) questions relating to iron deficiency assessment, and five (5) questions relating to iron treatment. This questionnaire will take approximately 15-20 minutes to complete.

Please note, for physiologists and researchers, please answer all questions pertaining to identifying iron deficienc iron treatment in the context of research only. A report will be provided to you concluding the study summarising the responses to each question.

Your survey data will be securely stored in the University of Canberra's Qualtrics server and treated with absolute confident In any manuscripts, reports, or other publications resulting from this study, you will not be able to be identified. In complian with the university protocol, all data collected from the surveys will be stored at the University of Canberra for five years ar destroyed.

- ◯ I agree that I am providing informed consent to participate in this project.
- ◯ I do not agree that I am providing informed consent to participate in this project (please close the web page).

Import from library Ac

Add Block

Demographics

Q1

1. Please indicate your gender.

- ◯ Female
- ◯ Male
- ◯ Non-binary / third gender
- ◯ Prefer not to say

Q2

2. What country do you reside in?

Q3

3. Please indicate your level of qualification.

- ◯ Provisional sports dietitian
- ◯ Accredited practicing dietitian
- ◯ Accredited sports dietitian
- ◯ Advanced accredited sports dietitian
- ◯ Fellow, Sports Dietitians Australia
- ◯ Other: ______

Q4

4. Please indicate the number of years you have spent practicing.

- ◯ <1 year
- ◯ 2-3 years
- ◯ 4-5 years
- ◯ 6-10 years
- ◯ 11+ years

Q5

5. What are the two sports you primarily work with (if you only work with one sport, please leave one response)?

- ☐ Name of sport 1 ______
- ☐ Name of sport 2 ______

Page Break

Q6

6. Please indicate the number of years you have spent working with your primary sport(s).

	${q://QID8/ChoiceTextEntryValue/4}	${q://QID8/ChoiceTextEntryValue/5}
<1 year	☐	☐
2-3 years	☐	☐
4-5 years	☐	☐
6-10 years	☐	☐
11+ years	☐	☐

Q7

7. Please indicate the level of athlete you primarily work with.

	${q://QID8/ChoiceTextEntryValue/4}	${q://QID8/ChoiceTextEntryValue/5}
World class (Olympic and/or world medallist):Right	☐	☐
Elite/ international (competing at an international level, team-sport athletes competing on a national team):Right	☐	☐
Highly trained/ national (competing at a national level, team-sport athletes competing in national and/or state/ tournaments):Right	☐	☐
Trained/ developmental (local level representation, regularly training ~3x.week):Right	☐	☐
Recreationally active (Meet WHO activity minimum activity guidelines [i.e., 150-300 min of moderate-intensity.week activity or 75-150 min of vigorous-intensity.week):Right	☐	☐

Q8

8. Please indicate the sex of the athletes you primarily work with.

	${q://QID8/ChoiceTextEntryValue/4}	${q://QID8/ChoiceTextEntryValue/5}
Female	☐	☐
Male	☐	☐
Both biological sexes	☐	☐
Non-binary / third gender	☐	☐
Prefer not to say	☐	☐

Import from library Ac

Add Block

Iron deficiency and assessment

Q9

9. Is iron deficiency prevalent in the athletes you work with? If you work with athletes of both biological sexes in either of y sports, please indicate if it's an issue with both biological sexes or just one.

	${q://QID8/ChoiceTextEntryValue/4}	${q://QID8/ChoiceTextEntryValue/5}
Yes	☐	☐
Females	☐	☐
Males	☐	☐
Females and males	☐	☐
No	☐	☐
Unsure	☐	☐

Q10

10. What level of importance do you place upon iron deficiency relevant to these other issues? Please order the following f
highest to lowest priority.

- Iron deficiency
- Macronutrient intake
- Micronutrient intake (excluding iron)
- Nutritional periodisation
- Total energy intake
- Other

Q11

11. What is the referral process for assessing an athlete's iron status in your sport? (e.g., team doctor, athlete GP).

Q12

12. How often do you assess your athlete's iron status?

- ○ Annually
- ○ Bi-annually
- ○ Quarterly
- ○ Only when an athlete has suspected iron deficiency
- ○ Other:

Q13

13. If an athlete presents as iron deficient, how often do you reassess their iron status until normal?

- ○ Monthly
- ○ Every 3 months
- ○ Every 6 months
- ○ Annually
- ○ Other:

Q14

14. Please indicate what blood markers you use to identify iron deficiency.

- ☐ C-reactive protein
- ☐ Haemoglobin concentration
- ☐ Haemoglobin mass
- ☐ Mean cellular haemoglobin
- ☐ Mean cellular volume
- ☐ Serum ferritin
- ☐ Serum soluble transferrin receptor
- ☐ Transferrin saturation
- ☐ Zinc protoporphyrin
- ☐ Other:

Q15

Q15. Do you utilise different markers to differentiate between the three stages of iron deficiency? If yes, what markers do y use?

- ◯ Yes ________
- ◯ No

Q16

16. The standard reference ranges for serum ferritin, soluble transferrin receptor, transferrin saturation, and haemoglobin concentration are listed below for each stage of iron deficiency (Australian Institute of Sport, 2022). After clicking on the pic identify the standard reference ranges for each stage, in the text box provided, please indicate whether your reference rang used to identify iron deficiency, iron deficiency non-anaemia, and iron deficiency anaemia deviate from those in each image (Y/N).

Stage	Typical Laboratory Profile	Serum ferritin	Haemoglobin concentration	Transferrin saturation	C
Stage I: Iron Deficient Non-anaemia [Stage 1 IDNA]	**Serum Ferritin <35µg/L**<br>Soluble Transferrin Receptor [sTfR] <2.5mg/L<br>Transferrin Saturation >16%<br>Haemoglobin [Hb] – women >120g/L<br>Haemoglobin [Hb] – men >135g/L				
Stage II: Iron Deficient Non-anaemia [Stage 2 IDNA]	Serum Ferritin <20µg/L<br>Soluble Transferrin Receptor [sTfR] <2.5mg/L<br>Transferrin Saturation <16%<br>Haemoglobin [Hb] – women >120g/L<br>Haemoglobin [Hb] – men >135g/L				
Stage III: Iron Deficiency Anaemia [IDA]	Serum Ferritin <12µg/L<br>Soluble Transferrin Receptor [sTfR] >2.5mg/L<br>Transferrin Saturation <16%<br>Haemoglobin [Hb] – women <120g/L<br>Haemoglobin [Hb] – men <135g/L				

Q17

17. If your reference ranges used to identify ID, IDNA, and IDA deviate from those in each image, what value do you use ar what factors affect your applied values (e.g., clinical experience, athlete type)?

	Serum ferritin	Haemoglobin concentration	Transferrin saturation	C
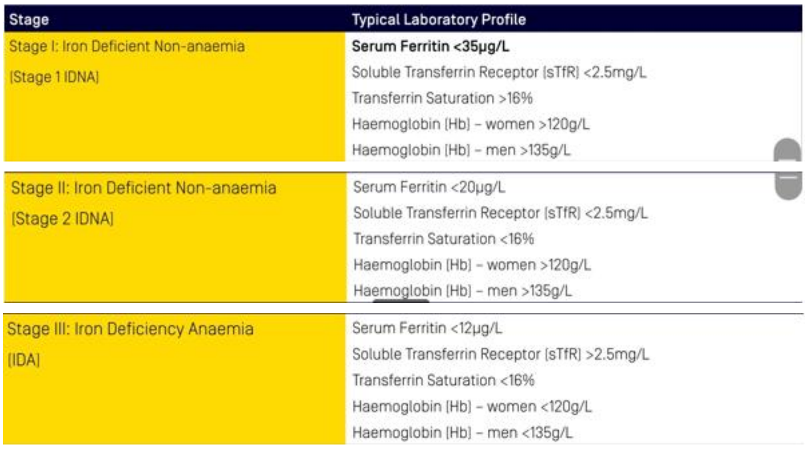				

Stage	Typical Laboratory Profile
Stage I: Iron Deficient Non-anaemia [Stage 1 IDNA]	**Serum Ferritin <35µg/L**<br>Soluble Transferrin Receptor [sTfR] <2.5mg/L<br>Transferrin Saturation >16%<br>Haemoglobin [Hb] – women >120g/L<br>Haemoglobin [Hb] – men >135g/L
Stage II: Iron Deficient Non-anaemia [Stage 2 IDNA]	Serum Ferritin <20µg/L<br>Soluble Transferrin Receptor [sTfR] <2.5mg/L<br>Transferrin Saturation <16%<br>Haemoglobin [Hb] – women >120g/L<br>Haemoglobin [Hb] – men >135g/L
Stage III: Iron Deficiency Anaemia [IDA]	Serum Ferritin <12µg/L<br>Soluble Transferrin Receptor [sTfR] >2.5mg/L<br>Transferrin Saturation <16%<br>Haemoglobin [Hb] – women <120g/L<br>Haemoglobin [Hb] – men <135g/L

Import from library Ac

Add Block

Iron treatment

Q18

18. What nutritional methods do you implement across the three stages of iron deficiency (e.g., dietary, supplementation)?

	Nutritional method
Stage 1 iron deficiency	
Stage 2 iron deficiency non-anaemia	
Stage 3 iron deficiency anaemia	

Q19

19. Do you consider the athlete's training schedule and meal composition when recommending the timing of supplement ingestion? If yes, what recommendations to do you provide to athletes (please enter responses in the text box provided)?

○ Yes

○ No

Q20

20. What are your current supplementation recommendations for each stage of iron deficiency (i.e., strategy, frequency, ar [if applicable])?

	Supplementation protocol
Stage 1 iron deficiency	
Stage 2 iron deficiency non-anaemia	
Stage 3 iron deficiency anaemia	

Q21

21. Do your supplementation recommendations vary according to different phases of training and competition (e.g., altitud
training load, off-season)? If yes, what factors affect your recommendations (please enter responses in the text box provide

- Yes
- No

Q22

22. What factors influence the type of supplement recommended (e.g., liquid, brand, tolerability, athlete preference)?

Import from library Ac

Add Block

End of Survey

We thank you for your time spent taking this survey.

Your response has been recorded.
